# MINI-GASTRIC BYPASS: DESCRIPTION OF THE TECHNIQUE AND PRELIMINARY RESULTS

**DOI:** 10.1590/0102-6720201700040009

**Published:** 2017

**Authors:** Elinton Adami CHAIM, Almino Cardoso RAMOS, Everton CAZZO

**Affiliations:** 1 Department of Surgery, Faculty of Medical Sciences, State University of Campinas - UNICAMP, Campinas; 2 Instituto Gastro-Obeso Center, São Paulo), SP, Brazil

**Keywords:** Bariatric surgery, Gastric bypass, Obesity, Bile reflux, Gastroenterostomy, Cirurgia bariátrica, Derivação gástrica, Obesidade, Refluxo biliar, Gastroenterostomia.

## Abstract

*****Background*** ****:**

In recent years, a surgical technique known as single-anastomosis gastric bypass or mini-gastric bypass has been developed. Its frequency of performance has increased considerably in the current decade.

*****Aim*** ****:**

To describe the mini-gastric bypass technique, its implementation and preliminary results in a university hospital.

*****Methods*** ****:**

This is an ongoing prospective trial to evaluate the long-term effects of mini-gastric bypass. The main features of the operation were: a gastric pouch with about 15-18 cm (50-150 ml) with a gastroenteric anastomosis in the pre-colic isoperistaltic loop 200 cm from the duodenojejunal angle (biliopancreatic loop).

*****Results*** ****:**

Seventeen individuals have undergone surgery. No procedure needed to be converted to open approach. The overall 30-day morbidity was 5.9% (one individual had intestinal obstruction caused by adhesions). There was no mortality.

*****Conclusion*** ****:**

Mini-gastric bypass is a feasible and safe bariatric surgical procedure.

## RESUMO

*****Racional*** ****:**

Nos últimos anos foi desenvolvida técnica cirúrgica conhecida como derivação gástrica com anastomose única ou mini-bypass gástrico. A frequência com que este procedimento vem sendo realizado aumentou consideravelmente na última década.

*****Objetivo*** ****:**

Descrever a técnica do procedimento, sua implementação e resultados preliminares em um hospital universitário.

*****Métodos*** ****:**

Estudo prospectivo em andamento para avaliar os efeitos em longo prazo do mini-bypass gástrico. As principais características do mini-bypass gástrico laparoscópico foram: uma bolsa gástrica com cerca de 15-18 cm (50-150 ml) com anastomose gastroentérica em alça isoperistáltica pré-cólica a 200 cm do ângulo duodenojejunal (alça biliopancreática).

Foram operados 17 pacientes. Nenhum procedimento necessitou conversão para laparotomia. A morbidade global em 30 dias foi de 5,9% (um caso de obstrução por brida). Não houve mortalidade.

*****Conclusão*** ****:**

O mini-bypass gástrico é procedimento cirúrgico bariátrico factível e seguro.

## INTRODUCTION

The prevalences of obesity and overweight have reached epidemic proportions in the last decades, with estimates by the World Health Organization (WHO) pointing that about 2 billion people are at least overweight worldwide^9^ ^,^ ^29^. 

Bariatric surgery has been performed with increasing frequency around the world over the last decades, especially because of the extremely superior results in relation to long-term sustained weight loss and resolution of comorbidities than those observed with non-surgical therapies^4^ ^,^ ^5^ ^,^ ^7^ ^,^ ^8^ ^,^ ^27^. The overall impact of bariatric surgery has been demonstrated, with reductions of 40% on the mortality for any cause, 56% for coronary disease, 92% for complications of diabetes, and 60% for any malignant neoplasia^1^.

After almost 50 years of evolution from the initial descriptions proposed by Mason and Ito^16^, the laparoscopic Roux-en-Y gastric bypass became one of the surgical procedures of reference for the treatment of morbid obesity. However, despite its recognized efficiency and safety, this procedure presents considerable technical difficulty, even for experienced surgeons with appropriate training^14^. 

In recent years, a surgical technique known as single-anastomosis gastric bypass (SAGB) or mini-gastric bypass (MGB) has been developed; its frequency of performance has increased considerably in the current decade.^26^ Initially described by Rutledge^26^, this procedure proposes a simplification of Roux-en-Y bypass by performing a single anastomosis, with a significant reduction of technical complexity, shorter operative time and a potential reduction in morbidity and mortality. Several studies have demonstrated the benefits provided by this procedure, including excess weight loss and resolution of comorbidities equivalent or even higher than those observed after the Roux-en-Y gastric bypass^3^ ^,^ ^6^ ^,^ ^10^ ^,^ ^12^ ^,^ ^13^ ^,^ ^17^ ^,^ ^19^ ^,^ ^21^ ^,^ ^23^ ^,^ ^25^ ^,^ ^28^.

This study aims to describe the mini-gastric bypass technique and its implementation and preliminary results in a university hospital.

## METHODS

This study was designed as a detailed description of a surgical technique and the preliminary outcomes obtained in the first operated cases. It is part of a larger ongoing trial evaluating the long-term effects of MGB which underwent evaluation and was approved by the local Ethics Research Board under the reference Unicamp/1.957.057. Bariatric surgery was warranted based on the National Institutes of Health consensus statement^20^and Brazilian Department of Health recommendations^18^. All individuals who took part in the study provided informed consent. All the procedures were performed by the same surgical team. The outcomes evaluated in the current study were: surgical time, estimated intra-operative bleeding, 30-day morbidity and mortality, hospital stay, and number of cartridges utilized.

### Positioning of the patient and the surgical team

The operation is performed with the patient in the supine position with legs open and with 45 degrees inclined position. The attachment of the patient to the surgical table is made ​​by placing two belts (on the abdominal area and below the level of the thighs, respectively). The surgeon stands between the legs, with the 1^st^ assistant, who handles the camera and the auxiliary clamp, and the scrub on the right. Urinary bladder catheterization is not used. Antibiotic prophylaxis were routinely administered. The prevention of thrombotic events is made ​​with use of graduated compression stockings and intermittent pneumatic boots. A disposable orogastric tube (Fouchet 32-Fr) is routinely placed.

### Pneumoperitonium e placement of the trocars

**FIGURE 1:**
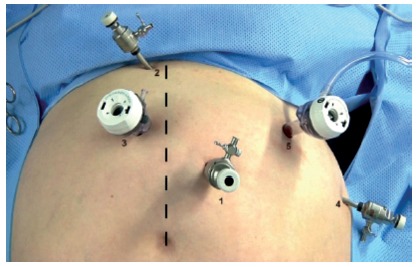
Placement of the trocars (Adapted from Ramos et al.^22^)

The pneumoperitoneum is performed by means of a direct puncture with a Veress needle in the left upper quadrant, near the costal margin at the level of the midclavicular line (Palmer’s point). The initial pressure is set at 15 mmHg, and maintained till the expected pressure (about 15 mmHg) is reached. The surgery initiates by the placement of the 10 mm permanent trocars for introduction of 30 degrees optics/camera placed at the mesogastrium between 12-15 cm below the xiphoid process and 3 cm to the left of the midline, considered as number 1 trocar. The trocar number 2, of 5 mm, is placed near the xiphoid process for the use of liver retractor which is usually a stick/probe held by the 2^nd^ assistant. The number 3, disposable of 12 mm, is used by the surgeon’s left hand, placed on the right side of the patient in an intermediate position between the previous two, 3-5 cm lateral to the midline. The number 4, also permanent of 5 mm, is placed along the left costal margin in the anterior axillary line to the 1^st^ assistant. The last trocar, number 5, disposable of 12 mm, is placed adjacent to the left costal margin in the hemi-clavicular line to surgeon’s right hand manipulation. The pneumoperitoneum is maintained by trocar number 5. Figure 1 presents the trocars’ placement^22^.

### Surgical Technique

**FIGURE 2:**
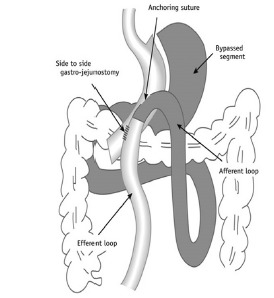
Schematic representation of the mini-gastric bypass (Adapted from Park et al.^24^)

The operation begins with the dissection of the esophagogastric angle and the opening of the left gastrophrenic ligament with a harmonic scalpel, so as to expose the lateral aspect of the left diaphragmatic crus. Then, the ressection of the fat pad of the esophagogastric junction (Belsey’s fat) is performed. Then, the surgeon proceeds the ligation of the distal lesser sac, next to the insertion of the Latarjet nerve, using a harmonic scalpel until the exposure of the posterior gastric wall. The gastric pouch must be lengthy and narrow, measuring around 15-18 cm, with a 50-150 ml reservoir capacity. The pouch is created using 01 unit of 45mm blue cartridges to perform the horizontal section and 02 to 03 units to perform the vertical section. The stapling lines of the pouch and excluded stomach are then reinforced with a 3-0 polydioxanone continuous suture. The Treitz ligament is then identified and the small bowel is counted until 200 cm from the Treitz angle, determining the exclusion of part of the stomach, duodenum, and proximal jejunum from the food pathway. This segment is then attached to the pouch and a vertical or slightly oblique omega-loop, isoperistaltic, antecolic, and side-to-side 25mm-gastrojejunostomy is performed using a 45mm white cartridge; the orifice for the cartridge insertion is closed by means of a continuous suture with 3-0 polydioxanone reinforced with separate stitches of 3-0 polyester. The Petersen’s defect is closed by means of a continuous suture with 3.0 silk^9^. The placement of a silicone ring around the gastric pouch is randomly opted following the study protocol for evalution of the effects of the ring. The randomization is performed by means of an electronic device and the individuals are notified of the result of the randomization process prior to the surgery. Among the individuals which have a 6.5-cm silicone ring placed, it is attached to the pouch with 3-0 polypropylene stitches. Figure 2 presents a schematic representation of the surgical technique.^24^

### Postoperative Protocol

All individuals which underwent surgery remained in a fasting state for 48 hours. Then, an oral methylene blue challenge and a contrast upper radiograph series are performed. Whether there is no evidence of leaks, oral diet is initiated and the individuals are discharged on postoperative day 03^9^.

## RESULTS

Until the submission of this study, 17 individuals have undergone surgery. No procedure needed to be converted to the open approach. The overall 30-day morbidity was 5.9% (one individual who presented with intestinal obstruction caused by adhesions). There was no mortality. The detailed results are presented in Table 1.

**TABLE 1:** Characteristics of the study group and early postoperative outcomes (n=17)

N	17
Gender	M: 4 (23.5%); F: 13 (76.5%)
Body mass index (kg/m2)	36.5 (35 -50.1)
Approach	Laparoscopic: 17 (100%)
Conversions to Open Approach	0
Operative time (minutes)	64.3 (45 - 120)
Estimated bleeding (mL)	14.7 (0 - 100)
Hospital stay (days)	3.1 (3 - 5)
Stapler cartridges per procedure	4.3 (4-5)
Readmissions	1 (5.9%) - Intestinal obstruction

## DISCUSSION

The major concern regarding the MGB technique is the potential risk of gastric and esophageal cancer due to the possibility of biliary reflux to the gastric pouch and gastroesophageal junction. However, a number of traits were added to this surgical technique since its inception, especially to minimize these risks^25^ ^,^ ^26^. The Mason loop gastric bypass and the Billroth II partial gastrectomy are the two procedures which at first sight seem to be very look alike the MGB^15^. Albeit based on the same premises, MGB presents substantial advances from the failures of these procedures. Firstly, the pouch, which is lengthier and narrower than that of the classic gastric bypass, is designed to understate the reflux of enteric secretion through the anastomosis. Secondly, the anastomosis itself, which is vertical or slightly oblique in the posterior wall of the pouch, favors the gastric emptying and potentially avoids significant reflux. Both Mason loop bypass and Billroth II gastrectomy were based on large horizontal pouches, which did not support the gastric emptying and also could facilitate the occurrence of gastric stasis, thus favoring the biliary reflux. Furthermore, the distance from the Treitz angle, composing a long biliopancreatic limb (around 200 cm) permits the resorption of large amounts of the biliary secretion; thus, the enteric juice that arrives at the anastomosis site is not so concentrated as the one which usually arrived at the Billroth II gastrectomy design^15^ ^,^ ^25^ ^,^ ^26^.

Besides these design issues, there are also other issues raised in relation to the carcinogenesis of biliary reflux. Based on *in vitro* and animal studies, it has been suggested that the reconstruction with a loop configuration in patients undergoing gastric bypass could increase the risk of gastric and esophageal cancers.^2^ ^,^ ^11^ However, although in the 1960s and 1970s thousands of Mason loop gastric bypasses have been performed, there was only a single case report of cancer in the gastric pouch following this surgery^2^; accordingly, even after tens of thousands of MGBs performed since 1997, there has been only one gastric cancer reported in a MGB patient - and it was in the bypassed stomach and not in the pouch^30^. Since the the overall annual incidence of both these cancers is estimated to be one case per 7,000-10,000, this historical data is significant^11^ ^,^ ^15^.

The current study revealed significant results in regards to early outcomes; MGB led to both early morbidity and mortality comparable to those observed after RYGB. Furthermore, due to its simplified design and the necessity to perform a single anastomosis, it is simpler and potentially more cost-effective, since less stapler cartridges are necessary.

The major limitations of this study are the small sample of individuals who underwent surgery and the short postoperative follow-up time; both these factors do no permit a thorough evaluation of the long-term risk-effectivity ratio and inferences on the late outcomes of this procedure. Nonetheless, since the main objective of this study was to show the feasibility of the procedure, further research and long-term follow-up are needed to provide more evidence in regards to its long-term outcomes.

## CONCLUSION

MGB is a feasible and safe bariatric surgical procedure.
